# Jordanian Population’s Perception and Understanding of Psoriasis: A Cross-Sectional Study

**DOI:** 10.7759/cureus.68977

**Published:** 2024-09-09

**Authors:** Rand Murshidi, Nour Shewaikani, Assem Al Refaei, Dana Q Khateeb, Raghad Al-Shami, Bayan E Hwidi, Maram Nasrallah, Leen Alshamasneh, Raghad Murshidi, Mahmoud Abdallat

**Affiliations:** 1 Department of Dermatology, Jordan University Hospital, Amman, JOR; 2 Department of Internal Medicine, School of Medicine, The University of Jordan, Amman, JOR; 3 Department of Dermatology, School of Medicine, The University of Jordan, Amman, JOR; 4 Department of Otolaryngology, Jordan University Hospital, Amman, JOR; 5 Department of Neurological Surgery, Jordan University Hospital, Amman, JOR

**Keywords:** attitudes, knowledge, perceptions, psoriasis, public health

## Abstract

Background

Psoriasis is a chronic immune-mediated disease with a 2-3% prevalence. As with other diseases with cutaneous manifestations, psoriasis patients suffer from psychological issues and social isolation. The general population’s misconceptions and prejudicial attitudes toward those patients are highly implicated in ensuing psychological issues. Accordingly, this study is the first to investigate the Jordanian population’s knowledge about and attitude toward psoriasis.

Methods

Our cross-sectional study was conducted using a self-administered online questionnaire that 1,306 participants from the Jordanian population completed. The questionnaire constituted four sections addressing the sociodemographic characteristics, previous exposure to psoriasis, knowledge about psoriasis, and attitudes toward psoriasis. The data analysis was conducted using R and RStudio packages.

Results

The averages and standard deviations of knowledge and attitude scores of the total sample were 7.54 ± 2.38 and 3.45 ± 9.22, respectively. When further classified into categories, 73.81% appeared in the moderate knowledge category. Moreover, 61.49% had a positive total attitude score. Among the common misconceptions identified were thinking that psoriasis is a hereditary disease (30.09%), denying that psoriasis increases the risk of diabetes (51.68%) and heart disease (67.69%), and not knowing that it affects the social life of patients (26.11%). Of the prominent negative attitudes observed were those related to the intimate relationship status and sharing a swimming pool with a psoriasis patient.

Conclusions

Although the Jordanian population’s overall knowledge level appeared sufficient, some critical misconceptions were identified. Moreover, this research revealed a high prevalence of negative attitudes toward psoriasis among the Jordanian public. Future research could link every negative behavior to their exact triggering misconception. This may further assist in the institutional effort to combat discriminatory behaviors.

## Introduction

Psoriasis is a chronic immune-mediated disease with an estimated global prevalence of 2-3% [1]. While it can appear at any age, psoriasis’s two onset peaks are at ages 20-30 and 50-60 [2]. Moreover, psoriasis is associated with psoriatic arthritis and may increase the risk of metabolic syndrome, chronic kidney disease, and cardiovascular diseases [3,4]. Multiple environmental and behavioral factors, such as skin trauma, infections, smoking, some drugs, such as lithium and interferon, and even stress, can trigger the onset of psoriasis in genetically susceptible individuals [5]. The degree of psoriasis knowledge and awareness in the community correlates with stigmatization or acceptance. Accordingly, the general population’s reactions to psoriasis patients may lead to social isolation, a decline in self-esteem and life satisfaction, and depression [6,7]. This relationship has been explored, revealing that both the inflammatory nature of the disease and stigma contribute to depression [6]. The incidence of depression keeps on growing, especially among severe psoriasis patients aged 40-50 among other inflammatory diseases [8]. Numerous studies have shown a severe lack of understanding and negative attitudes toward people who have psoriasis [9,10]. According to a study from Jordan about the perception and understanding of common skin disorders, most respondents (63.8%) agreed that having psoriasis impacts a patient’s quality of life and makes it uncomfortable [11]. The high level of stigmatization in the region was further evidenced during the COVID-19 pandemic, when 64% of respondents expressed stigma toward infected individuals [12]. A study from Germany showed that only 29% of participants were familiar with the term “psoriasis” [13]. Furthermore, according to a survey of nonmedical students at Qassim University in Saudi Arabia, 13.5% of respondents did not know about psoriasis [14].

The primary objective of this study is to assess the level of knowledge and attitudes toward psoriasis among the Jordanian population. By identifying the gaps in knowledge and understanding the prevailing attitudes, this study seeks to inform public health initiatives aimed at reducing stigma and improving the quality of life for psoriasis patients in Jordan.

This article was previously posted in the Research Square preprint server on April 7, 2023.

## Materials and methods

Study design

Our cross-sectional study was conducted using a self-administered online questionnaire completed by 1,306 participants from the Jordanian population between November 2022 and February 2023. A dermatologist and a group of statisticians designed the survey with some adopted questions from published similar research [12,13]. The survey included four sections; the first one addressed the participants’ sociodemographic characteristics, including age, gender, educational level, marital status, geographical distribution, household income, employment status, and whether they have a health-related profession. The second section was designed to capture the participants’ previous exposure to psoriasis using four questions: if they have heard about psoriasis before, were diagnosed with psoriasis, knew someone diagnosed with psoriasis, or lived with someone diagnosed with psoriasis. The third section included 15 questions to assess the participant’s knowledge of the disease, with yes, maybe, and no answers. Lastly, the fourth section assessed participants’ attitudes toward psoriasis and constituted 10 statements using the 5-Point Likert Scale to evaluate their agreement with them. In the “knowledge” section, participants received one point for each correct answer, while incorrect answers and “maybe” responses were assigned zero points. In the attitude section, each of the five possible responses was scored between (-2) and (+2), with “strongly agree” scored as (-2), “neutral” as (0), and “strongly disagree” as (+2). The total score for each participant was calculated by summing their responses. In addition to analyzing the continuous knowledge and attitude scores, participants were categorized into three knowledge levels: low (0-5 points), moderate (6-10 points), and high (11-15 points) to better assess community knowledge about psoriasis. Attitudes were classified into two categories: positive (1-20) and negative (-20 to 0).

The minimum required sample size was 385, calculated using the Raosoft sample size calculator, assuming a population size of 11,000,000, a 5% margin of error, a 95% confidence interval, and 50% as the response distribution. Before starting with the data collection, a pilot study of 50 participants was conducted to assess the validity of the questionnaire. Afterward, data collection started by distributing the questionnaire on social media platforms, including Facebook, WhatsApp, Twitter, and Instagram. The questionnaire was completed anonymously without any identifiers being used to maintain the participants’ privacy throughout the process. A consent form, viewed on the first page of the questionnaire, was obtained from all participants (if the participants agreed to participate, they were asked to click “Start the questionnaire”).

Ethical approval for this study was obtained from the Institutional Review Board at Jordan University Hospital, in accordance with the ethical principles outlined in the Declaration of Helsinki.

Data analysis

Data were extracted from the electronic questionnaire and put into a Microsoft Excel spreadsheet (Microsoft Corporation, Redmond, WA, United States). Following that, data analysis was conducted through R [14] and RStudio [15]. Packages that were utilized include tidyverse for data wrangling [16], ggplot2 and ggpubr for plotting [17,18], rstatix for inferential analysis [19], and apaTables for the regression tables [20]. First, descriptive analysis was conducted to generate the counts and proportions of each variable. Then, the Shapiro-Wilk test was used to check the normality of our continuous variables (knowledge and attitude scores). Accordingly, Mann-Whitney and Kruskal-Wallis tests were used to run the inferential analysis. Finally, multiple linear regression was utilized to identify independent predictors of knowledge and attitude scores.

## Results

Respondents’ knowledge and attitude scores and sociodemographic characteristics influence on them

**Figure 1:**
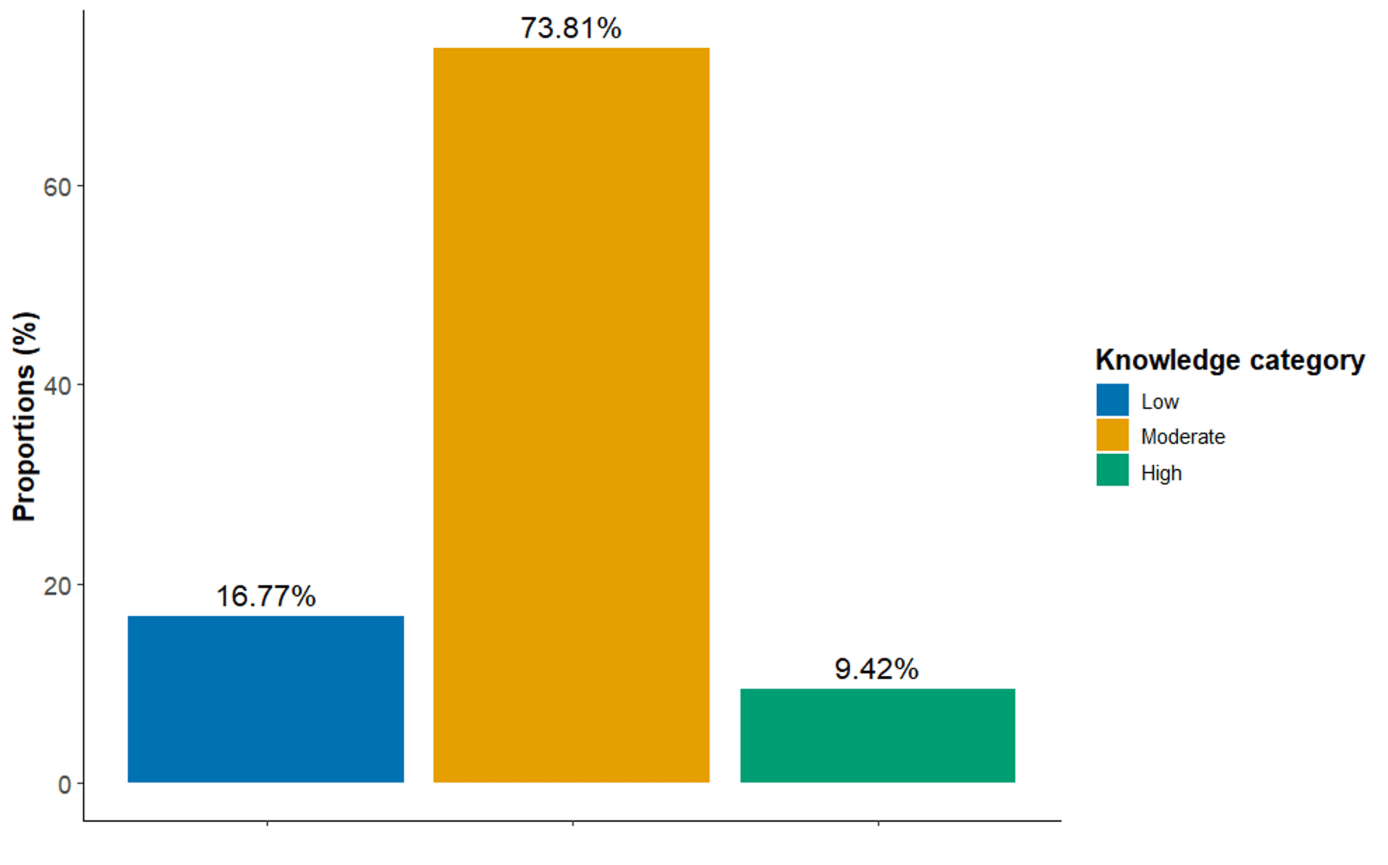
Proportions of respondents’ knowledge levels

A total of 1,306 individuals responded to the online questionnaire with an average and standard deviation of knowledge and attitude scores of 7.54 ± 2.38 and 3.45 ± 9.22, respectively. As shown in Figure 1, 964 (73.81%) of the respondents had moderate knowledge about psoriasis, while 123 (9.42%) were highly knowledgeable. Moreover, 803 (61.49%) had a positive total score of attitude toward psoriasis. About half (N = 709) of the respondents were between 16 and 30 years of age and showed significantly higher attitude scores when compared to other age groups (p < 0.001, and η2 = 0.01). Females represented 70% (N = 914) of our sample and had significantly higher knowledge and attitude scores (p = 0.001 and Cohen’s d = 0.09, and p < 0.001 and Cohen’s d = 0.12, respectively). Furthermore, 880 (67.38%) of respondents were from Jordan’s capital, Amman. According to the Kruskal-Wallis test, individuals living in the South had significantly higher knowledge scores but not attitude scores. Meanwhile, a significant association between marital status and attitude score was shown, but not with knowledge. Specifically, single respondents (N = 653, 50%) had higher scores when compared to married counterparts (N = 623, 47.7%) (p = 0.004). Table 1 presents the counts and proportions of each sociodemographic category, along with the corresponding knowledge and attitude analysis.

**Table 1:** Study respondents’ sociodemographic characteristics and related knowledge and attitude scores (N = 1,306) The data has been represented as N, %, mean, and standard deviation. A significant p-value was set as <0.05.

Variables		n	%	Knowledge scores				Attitude scores			
				Mean	SD	p-value	Effect size	Mean	SD	p-value	Effect size
Age group (years)	16-30 years	709	54.29	7.22	2.56	<0.001	0.014	4.29	9.81	<0.001	0.01
	31-50 years	434	33.23	7.91	2.13			2.43	8.74		
	>50 years	163	12.48	7.91	1.94			2.5	7.29		
Gender	Male	369	28.25	7.14	2.53	0.001	0.09	1.72	9.27	<0.001	0.12
	Female	937	71.75	7.7	2.29			4.14	9.11		
Income level	Less than 600 JD	435	34.69	7.62	2.39	0.003	0.007	2.74	9.23	0.047	0.003
	600-1,200 JD	570	43.64	7.27	2.3			3.48	9.08		
	More than 1,200 JD	283	21.67	7.8	2.42			4.53	9.41		
Education level	High school	67	5.13	6.85	2.41	0.014	0.005	1.72	9.68	0.061	0.003
	Bachelor	874	66.92	7.53	2.45			3.74	9.29		
	Diploma	193	10.11	7.48	2.22			2.18	8.61		
	Higher education	172	13.17	7.92	2.07			4.08	9.21		
Health profession	Yes	451	34.53	8.29	2.29	<0.001	0.229	4.96	9.61	<0.001	0.13
	No	855	65.47	7.14	2.32			2.66	8.91		
Occupation	Unemployed	305	23.35	7.56	2.22	<0.001	0.01	3.3	8.88	0.002	0.008
	Employed	590	45.18	7.82	2.19			2.69	9.04		
Geography	Student	411	31.47	7.12	2.67			4.65	9.61		
	North	67	5.13	7.01	2.86	0.008	0.006	3.18	9.69	0.181	0.001
	Amman	874	66.92	7.55	2.39			3.5	9.12		
	Center	193	10.11	7.44	2.11			4.32	9.04		
	South	172	13.17	8.11	2.11			1.87	9.7		

Influence of previous exposure to psoriasis on knowledge and attitude scores

Of the respondents, 261 (20%) had not heard of psoriasis before, and only 39 (3%) reported being diagnosed with the condition. The Mann-Whitney test showed a significant association between participants’ knowledge and attitude and whether they were aware of or lived with someone with psoriasis (p < 0.05). A summary of these results can be found in Table 2.

**Table 2:** Study respondents’ previous exposure to psoriasis and related knowledge and attitude scores (N = 1,306) A significant p-value was set as <0.05.

Variables	n	%	Knowledge scores				Attitude scores			
			Mean	SD	p-value	Effect size	Mean	SD	p-value	Effect size
Respondent has psoriasis	44	3.37	7.82	2.01	0.406	0.023	6	10.91	0.035	0.058
Respondent knows a patient with psoriasis	468	35.83	8.1	2	<0.001	0.171	5.68	8.78	<0.001	0.189
Respondent lives with a patient with psoriasis	104	7.96	8.17	1.91	0.002	0.082	8.09	9.44	<0.001	0.155
Respondent heard about psoriasis before	1052	80.55	7.87	2.14	<0.001	0.25	4.3	9.08	<0.001	0.192

Respondents’ sources of knowledge about psoriasis and its impact on their knowledge and attitude scores

**Figure 2:**
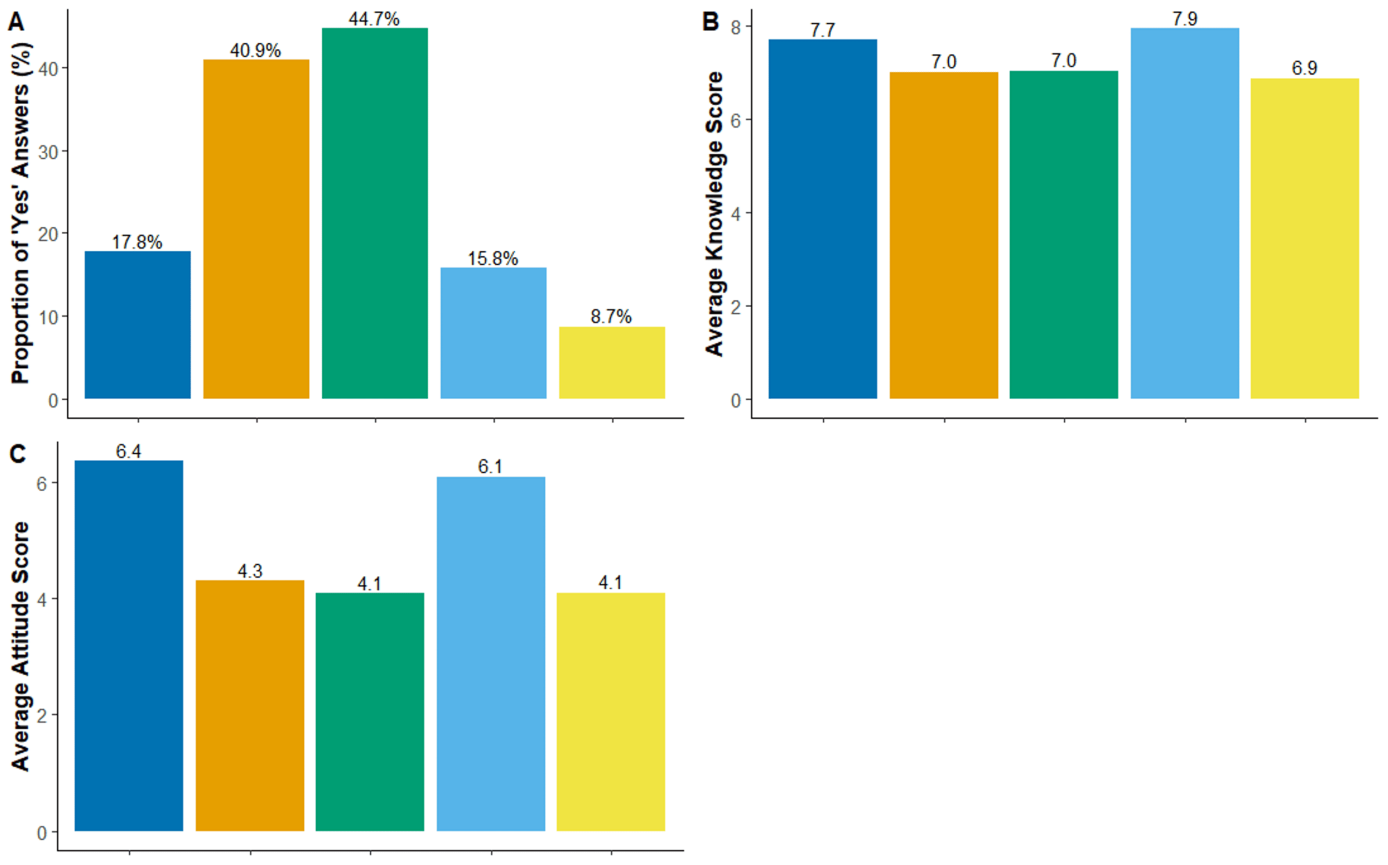
Sources of knowledge about psoriasis and their impact on knowledge and attitude scores

The results presented in Figure 2A indicate that a substantial proportion of respondents acquired knowledge about psoriasis from the internet and social media (N = 584, 44.72%) or from family and friends (N = 534, 40.89%). The Kruskal-Wallis test determined a significant difference in respondents’ knowledge and attitude scores with different knowledge sources (p < 0.001, and η2 = 0.02, and p = 0.02, and η2 = 0.007, respectively). Subsequent pairwise Wilcoxon rank-sum test revealed a significant trend of increased knowledge and attitude scores in respondents with knowledge sources that are either physicians or books and magazines when compared to respondents with other sources (p < 0.05, Figure 2B, 2C).

Regression analysis identified independent predictors of knowledge and attitude scores

The results of multiple linear regression analysis with knowledge score as the criterion variable revealed the following significant predictors: (1) male sex (beta = -0.39, p = 0.008); (2) ages between 31 and 50 years and ages above 50 years (beta = 0.45, p = 0.025, and beta = 0.62, p = 0.02, respectively); (3) diploma education (beta = -0.49, p = 0.01), (4) student (beta = -0.52, p = 0.006); (5) geographical location that is South Jordan (beta = 0.48, p = 0.024); (5) health profession (beta = 0.93, p < 0.001); (6) knowing a patient with psoriasis (beta =0.39, p = 0.008); (7) hearing before about psoriasis (beta = 1.12, p < 0.001); (8) physician as a source of knowledge (beta = 0.48, p = 0.011); and (9) attitude score (beta = 0.04, p < 0.001). The model explained 20.23% of the variance in the knowledge scores, and the F-test validated the fitness (F-statistic: 12 on 27 and 1278 degrees of freedom, p < 0.001).

On another hand, the results of multiple linear regression analysis with attitude score as the criterion variable revealed the following significant predictors: (1) male sex (beta = -2.00, p < 0.001); (2) ages between 31 and 50 years, and older than 50 years (beta = -2.30, p =0.005, and beta = -2.85, p = 0.008, respectively); (3) being from South Jordan (beta = -2.67, p = 0.002); (4) the student (beta = 1.70, p = 0.025); (4) knowing a patient with psoriasis (beta = 2.30, p < 0.001); (5) living with a patient with psoriasis (beta = 2.87, p = 0.006); (6) hearing before about psoriasis (beta = 1.89, p = 0.015); (7) books and magazines as a source of knowledge (beta = 2.24, p = 0.001); and (8) knowledge score (beta = 0.58, p < 0.001). The model explained 14.00% of the variance in the attitude scores, and the F-test validated the fitness (F-statistic: 7.706 on 27 and 1278 degrees of freedom, p < 0.001).

Respondents’ answers to the questions that constitute the knowledge and attitude scores

Of our sample, 165 (12.63%) reported that psoriasis is a contagious disease. Moreover, about half of the respondents denied or were not sure about psoriasis' autoimmune etiology. When asked about the role of magic or witchery in psoriasis, 59 (4.52%) believed that magic and witchery could trigger psoriasis. Table 3 presents the breakdown of respondents’ answers to questions assessing knowledge score.

**Table 3:** Respondents’ responses to questions regarding the knowledge of psoriasis The data has been represented as counts and percentages, N (%). * Correct answer

Questions		Count	%
Is psoriasis contagious?	No^*^	886	67.84
	Maybe	255	19.53
	Yes	165	12.63
Is psoriasis a hereditary disease?	No^*^	441	33.77
	Maybe	472	36.14
	Yes	393	30.09
Is psoriasis an autoimmune disease?	No	302	23.12
	Maybe	413	31.62
	Yes^*^	591	45.25
Does psoriasis affect the skin?	No	29	2.22
	Maybe	79	6.05
	Yes^*^	1198	91.73
Does psoriasis affect nails?	No	231	17.69
	Maybe	354	27.11
	Yes^*^	721	55.21
Does psoriasis affect joints?	No	349	26.72
	Maybe	418	32.01
	Yes^*^	539	41.27
Does psoriasis increase the risk of diabetes?	No	675	51.68
	Maybe	451	34.53
	Yes^*^	180	13.78
Does psoriasis increase the risk of heart disease?	No	884	67.69
	Maybe	338	25.88
	Yes^*^	84	6.43
Does psoriasis cause skin cancer?	No^*^	472	36.14
	Maybe	552	42.27
	Yes	282	21.59
Does psoriasis lead to death?	No^*^	984	75.34
	Maybe	261	19.98
	Yes	61	4.67
Is psoriasis caused by certain foods?	No^*^	827	63.32
	Maybe	338	25.88
	Yes	141	10.8
Is psoriasis triggered by psychological stress?	No	263	20.14
	Maybe	430	32.92
	Yes^*^	613	46.94
Is psoriasis triggered by magic or witchery?	No^*^	1063	81.39
	Maybe	183	14.01
	Yes	59	4.52
Does psoriasis affect the social life of patients?	No	341	26.11
	Maybe	394	30.17
	Yes^*^	571	43.72
Is there a treatment for psoriasis?	No	231	17.69
	Maybe	403	30.86
	Yes^*^	672	51.45

Regarding respondents’ attitudes toward psoriasis, 244 (18.68%) strongly agreed with the statement “I would not marry a psoriasis patient,” whereas only 115 (8.81%) strongly disagreed. On another note, a quarter of our sample (N = 338) strongly disagreed with the statement “I would want to divorce if my partner got diagnosed with psoriasis,” whereas only 51 (3.91%) of them strongly agreed. Table 4 presents the breakdown of respondents’ extent of agreement with statements assessing attitude scores.

**Table 4:** Respondents’ responses to questions regarding the attitudes toward psoriasis The data has been represented as counts and percentages, N (%).

Statements	Strongly disagree	Disagree	Neutral	Agree	Strongly agree
I would not eat food prepared by a psoriasis patient.	191 (14.62)	316 (24.20)	326 (24.96)	276 (21.13)	197 (15.08)
I would not eat at a shared table with a psoriasis patient.	309 (23.66)	445 (34.07)	256 (19.60)	193 (14.78)	103 (7.89)
I would not swim in a pool with a psoriasis patient.	178 (13.63)	281 (21.52)	309 (23.66)	305 (23.35)	233 (17.84)
I would not share my home with a psoriasis patient.	276 (21.13)	438 (33.54)	296 (22.66)	196 (15.01)	100 (7.66)
I would not marry a psoriasis patient.	115 (8.81)	224 (17.15)	439 (33.61)	284 (21.75)	244 (18.68)
My intimate relationship would be affected by my partner’s psoriasis diagnosis.	104 (7.96)	264 (20.21)	468 (35.83)	288 (22.05)	182 (13.94)
I would want to divorce if my partner got diagnosed with psoriasis.	338 (25.88)	508 (38.90)	336 (25.73)	73 (5.59)	51 (3.91)
I would not share my workplace with a psoriasis patient.	399 (30.55)	530 (40.58)	262 (20.06)	59 (4.52)	56 (4.29)
I would not shake hands with a psoriasis patient.	291 (22.28)	403 (30.86)	317 (24.27)	197 (15.08)	98 (7.5)
I would not hire a psoriasis patient.	409 (31.32)	498 (38.13)	244 (18.68)	90 (6.89)	65 (4.98)

## Discussion

Research has shown that skin-related disorders impose psychological burdens and socialization challenges on patients. Psoriasis appeared no exception, as studies elucidated higher depression and consequent isolation among psoriatic patients [6]. The general population’s misconceptions and prejudicial attitudes toward vulnerable patients are highly implicated in those issues [10]. Accordingly, due to the lack of studies assessing where Jordanians stand in this respect, this study is the first to investigate the knowledge about and attitude toward psoriasis among the Jordanian population. Based on our results, future directions in combating stigmatization toward psoriasis patients would be with a background of the common triggers and misconceptions in our society.

Our results revealed significant discriminatory attitudes among the Jordanian population toward psoriasis, as 38.51% of the respondents had more prevalent negative attitudes than positive ones. Similarly, a study in Malaysia reported 86% as the percentage of participants who reported one or more discriminatory practices toward psoriasis [8]. Among the observed negative attitudes in our study were not marrying a psoriasis patient (40.43%), affecting intimate relationships in case of a partner’s psoriasis diagnosis (36%), and wanting a divorce in case of a partner’s psoriasis diagnosis (9.5%). Around a third of the respondents also did not disagree but instead showed neutral feelings toward those attitudes. We view these results as highly concerning as they render the patients constantly anxious about their partner’s reluctance to maintain their relationship. This anxiety may fire back on their previously stable relationship; for instance, one study showed that wives’ daily anxiety would affect both partners’ perception of the relationship quality [21]. Moreover, Grundstrom et al.’s findings supported the evidence that being single or divorced carries higher risks of depressive symptoms and lower self-esteem [22]. Another facet of the problem is the anxiety and stress effect on the disease course. Literature shows that psychological stress may increase the recurrence and severity of psoriasis and could affect the treatment outcomes [23,24]. Inline appeared the results of a Saudi study, where 70% would not approve of their daughter’s marriage to a psoriasis patient [11]. Additionally, 38.82% of respondents disagreed with the statement about refusing to eat food prepared by psoriasis patients, while 35.15% disagreed with the idea of not sharing a table with them during meals [11]. Consistently, a German study concluded that 69% and 59% of the participants said that most people think psoriasis patients are disgusting and do not want to touch them, respectively [7]. Those results are alarming and need actions to combat them, and attributing them to the triggering misconception in future studies may serve in this.

Our regression showed that younger ages predicted a higher prevalence of positive attitudes despite their lower knowledge, which should be viewed optimistically for less discriminatory future generations. Additionally, lower knowledge was an independent predictor of more prevalent negative attitudes. This finding along, with the insufficient knowledge that psoriasis is not hereditary (33.77%), may explain the negative attitude in intimate relationships discussed previously. Most of our respondents have moderate knowledge about the disease (73.81%), and 16.77% are in the low knowledge category. For instance, 12.63% believed psoriasis is contagious, compared to 16.5% of a French study sample [12]. Furthermore, most respondents denied that psoriasis is a systemic inflammatory condition with comorbidities like diabetes (51.68%) and cardiovascular disease (67.69%), indicating that they merely view it as a cosmetic issue. The case was similar in the joint and nail involvement knowledge, where 26.72% and 17.69% denied it, and 32.0% and 27.11% said maybe, respectively. This misperception of the disease needs to be targeted by campaigns to deliver that it is an overly active immune system that can attack multiple body areas [25]. Meanwhile, 20.14% and 32.92% of the participants disagreed with the disease being triggered by psychological stress and answered maybe, respectively. Raising public awareness in these two aspects may lead to more acceptance and compassionate attitudes toward psoriasis patients.

We encourage utilizing predictors of higher knowledge levels in directing future efforts to combat discrimination toward psoriasis, including hearing about psoriasis and physicians as a source of knowledge. This could be achieved by inviting physicians to spread accurate medical knowledge and their perspectives about the psychological well-being of the patients. Moreover, future research could address the exact misconceptions that lead to the respondent’s negative behavior to further assist institutional efforts in combating prejudicial attitudes and behaviors. A limitation of our study is that the use of a self-administered online questionnaire may have introduced selection bias, potentially excluding individuals who are either unfamiliar with or lack access to the necessary technology. This limitation could skew the respondent pool toward individuals who are more comfortable with digital platforms, potentially overrepresenting younger or more technologically literate participants. As a result, our findings may lack generalizability to broader populations, particularly older adults or those in lower socioeconomic groups who may have limited internet access or digital proficiency. Future studies should consider using alternative data collection methods, such as face-to-face interviews or telephone surveys, to ensure a more inclusive sample and reduce potential biases introduced by technology-dependent methodologies.

## Conclusions

Although our study demonstrated that the overall knowledge level about psoriasis was generally sufficient, it also identified critical misconceptions that persist among the public. Furthermore, the research highlighted a high prevalence of negative attitudes toward psoriasis within the Jordanian population. A key limitation of this study is the potential selection bias introduced by the use of a self-administered online questionnaire, which may limit the generalizability of our findings. Future research should aim to link each negative behavior to its specific underlying misconception and employ more inclusive data collection methods. These efforts could provide valuable insights to further support institutional strategies in combating discriminatory behaviors and promoting awareness.
